# Extracellular signal-regulated kinase (ERK) activation is required for itch sensation in the spinal cord

**DOI:** 10.1186/1756-6606-7-25

**Published:** 2014-04-03

**Authors:** Ling Zhang, Guan-Yu Jiang, Ning-Jing Song, Ying Huang, Jia-Yin Chen, Qing-Xiu Wang, Yu-Qiang Ding

**Affiliations:** 1 Key Laboratory of Arrhythmias, Ministry of Education of China, East Hospital, Tongji University School of Medicine, Shanghai 200120, China; 2 Department of Anatomy and Neurobiology, Tongji University School of Medicine, Shanghai 200092, China; 3 Department of Dermatopathology, Shanghai Skin Disease Hospital, Shanghai 200443, China; 4 Department of Anesthesiology, East Hospital, Tongji University School of Medicine, Shanghai 200120, China; 5 Department of Dermatology, Shanghai International Medical Center, Shanghai 201318, China

**Keywords:** Acute itch, Chronic itch, pERK, H1 receptor, Spinal cord, Atopic dermatitis

## Abstract

**Background:**

Itch, chronic itch in particular, can have a significant negative impact on an individual’s quality of life. However, the molecular mechanisms underlying itch processing in the central nervous system remain largely unknown.

**Results:**

We report here that activation of ERK signaling in the spinal cord is required for itch sensation. ERK activation, as revealed by anti-phosphorylated ERK1/2 immunostaining, is observed in the spinal dorsal horn of mice treated with intradermal injections of histamine and compound 48/80 but not chloroquine or SLIGRL-NH2, indicating that ERK activation only occurs in histamine-dependent acute itch. In addition, ERK activation is also observed in 2, 4-dinitrofluorobenzene (DNFB)-induced itch. Consistently, intrathecal administration of the ERK phosphorylation inhibitor U0126 dramatically reduces the scratching behaviors induced by histamine and DNFB, but not by chloroquine. Furthermore, administration of the histamine receptor H1 antagonist chlorpheniramine decreases the scratching behaviors and ERK activation induced by histamine, but has no effect on DNFB-induced itch responses. Finally, the patch-clamp recording shows that in histamine-, chloroquine- and DNFB-treated mice the spontaneous excitatory postsynaptic current (sEPSC) of dorsal horn neurons is increased, and the decrease of action potential threshold is largely prevented by bathing of U0126 in histamine- and DNFB-treated mice but not those treated with chloroquine.

**Conclusion:**

Our results demonstrate a critical role for ERK activation in itch sensation at the spinal level.

## Background

Itching is an unpleasant cutaneous sensation that provokes the desire or reflex to scratch. The sensation of itch can be divided into acute and chronic categories, according to its persistence period [1,2]. Acute itch, much like acute pain, serves as a physiological, self-protective warning signal that helps prevent the body from being hurt. Chronic itch, however, is a common symptom of many serious dermatological illnesses, as well as diseases affecting other body systems. In the case of chronic itch, anti-histamines are usually not an effective clinical treatment [3,4]. A better understanding of the cellular and molecular mechanisms that underlie different itching sensations and their processing is urgently needed in order to develop new itch relief methods.

In the last five years, huge progress has been made towards elucidating the mechanisms underlying itch development at the spinal level. In particular, gastrin releasing peptide receptor (GRPR)-expressing neurons in the spinal dorsal horn have been found to mediate both histamine-dependent and -independent acute itch, but are not involved in pain processing [5,6]. Moreover, the G-protein-coupled receptor MrgprA3 was shown to mediate the itch response induced by chloroquine, an anti-malarial drug defined as a histamine-independent pruritogen, but not those responses induced by histamine [7]. Mice lacking the transcription factor *Bhlhb5* in the spinal cord display enhanced spontaneous scratching behaviors, but their pain behaviors remain unchanged [8]. Toll-like receptor 3 and 7, the family members that mediate innate immunity, are important for the development of itch and spinal synaptic transmission [9,10]. Furthermore, mice in which vesicle glutamate transporter type 2 expression has been specifically deleted from Nav1.8-expressing primary sensory neurons in the dorsal root ganglia (DRG) exhibit not only decreased glutamate release in the spinal dorsal horn, but also decreased pain sensation and enhanced itch responses [11].

Extracellular signal-regulated kinases ERK1/2, two closely related members of the mitogen-activated protein kinase family, transduce extracellular stimuli into intracellular signaling through various transcriptional and post-translational mechanisms [12]. Accumulating evidence has shown that noxious peripheral stimulation promotes the phosphorylation of ERK1/2 (pERK) in the spinal dorsal horn, and that ERK1/2 activation is important for the development of inflammatory and neuropathic pain via the enhancement of synaptic plasticity in the spinal cord [13-15]. Pain and itch are the two major somatosensations, which are transmitted by primary sensory neurons in the DRG, processed in the spinal dorsal horn first and then conveyed by the second-order neurons in the spinal dorsal horn to the supraspinal sites for the generation of individual perception [16,17]. Given that pain and itch have much in common, in the present study, we set out to explore whether ERK activation is involved in processing itch-related signals in the spinal cord.

## Results

### Spinal ERK signaling is activated in histamine- but not chloroquine-induced acute itch

**Figure 1:**
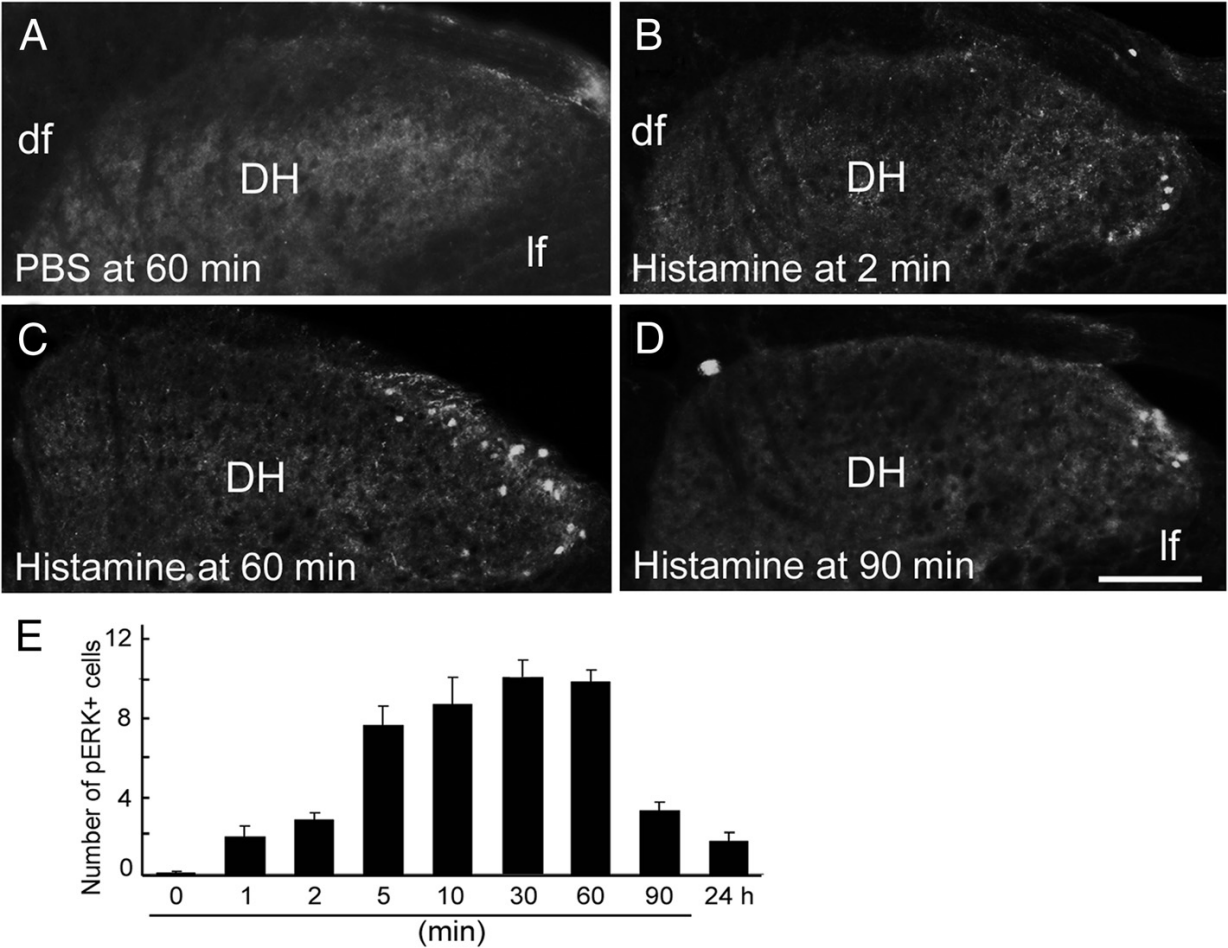
**Time-dependent induction of pERK expression by histamine. (A)** Few pERK-positive cells are seen in PBS-injected mice. **(B-E)** An obvious increase in the number of pERK-positive cells is observed at 2 min post-histamine injection **(B, E)**, peak levels are achieved at 30 min and sustained until 60 min **(C, E)**, and a marked decline is seen at 90 min post-injection **(D, E)**. *n* = 6 for each time point. DH, dorsal horn; df, dorsal fasciculus; lf, lateral fasciculus. Scale bar, 100 μm.

**Figure 2:**
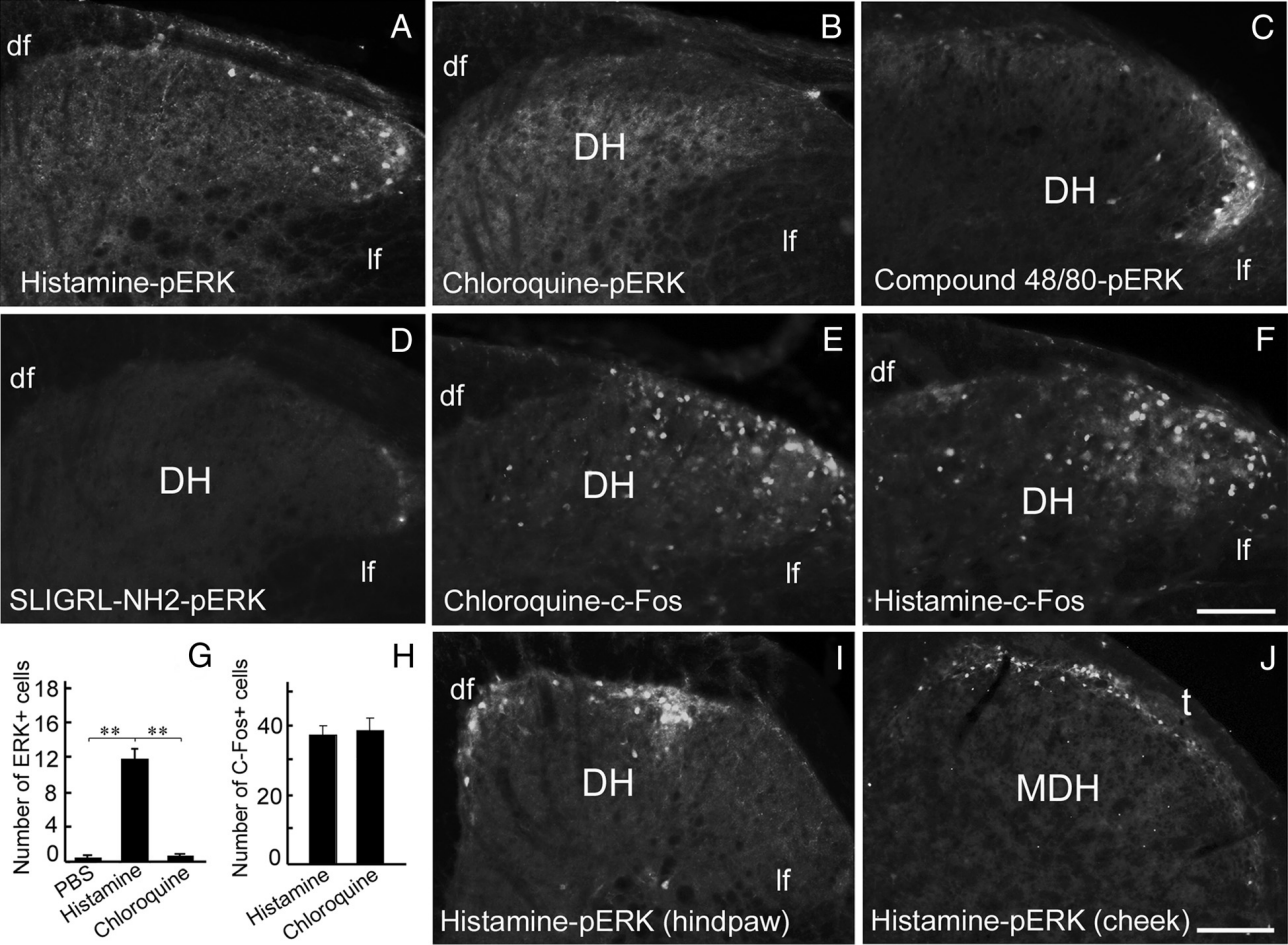
**Expression of pERK and c-Fos in the spinal dorsal horn 30 min after injection of histamine, compound 48/80, chloroquine or SLIGRL-NH2. (A-D)** pERK is expressed in the lateral dorsal horn of mice injected with histamine and compound 48/80 **(A, C)** but not with chloroquine and SLIGRL-NH2 **(B, D)** in the nape. **(E, F)** Similar c-Fos expression is observed in the dorsal horn after injection of chloroquine or histamine in the nape. **(G)** Comparison of the number of pERK-positive cells in the dorsal horn of mice treated with PBS, histamine and chloroquine. One way ANOVA; *P* < 0.001; **P < 0.01; *n* = 5 for each group. **(H)** Comparison of the number of c-Fos-positive cells in the dorsal horn of mice treated with chloroquine or histamine as shown in **(E, F)**. Student’s t-test; *P >* 0.05; *n* = 5 for each group. **(I)** pERK is expressed in the medial dorsal horn of mice that received histamine injection in a hind paw. **(J)** pERK-positive cells are distributed in the dorsal part of the medullary dorsal horn of mice with histamine injection in the cheek region. DH, dorsal horn; df, dorsal fasciculus; lf, lateral fasciculus; MDH, medullary dorsal horn; t, spinal trigeminal tract. Scale bars, 100 μm in **F** (applies to **A**-**F** and **I**) and 200 μm in **J**.

Histamine and chloroquine were used to induce histamine- and non-histamine-dependent acute itch, respectively [5]. Consistent with previous reports [5,7,9], intradermal injections of histamine into the nape elicited vigorous scratching with the hind paws within the first 30 min (201.60 ± 39.69/30 min). Scratching decreased greatly after this time point, and thus behavioral data from the first 30 min post-injection were used for comparison. Since pERK is an indicator of ERK activation [13,18,19], we examined its expression in the spinal cord at various time points after histamine injection. The number of pERK-expressing cells was obviously increased as of 5 min post-injection, peaked at 30 min, maintained peak levels until 60 min, then decreased dramatically at 90 min relative to controls received PBS injection (Figures 1 and 2A, G). In addition, pERK-positive cells were also observed in anesthetized mice that did not scratch, showing that the ERK activation is caused by histamine rather than scratching itself. Taken together, these results demonstrate that injection of histamine leads to ERK activation in the spinal cord.

Consistent with previously reported findings [5-7], chloroquine also induced vigorous scratching responses (220.16 ± 35.75/30 min), but did not lead to an obvious increase of pERK expression in the spinal cord at any of the time points examined (one way ANOVA, *P* > 0.05), as compared with mice injected with PBS (Figures 1A and 2B, G). Therefore, ERK signaling in the spinal cord is specifically activated in histamine-induced, but not chloroquine-induced acute itch.

To further confirm the specific activation of ERK signaling in histamine-induced itch, we tested two other substances: compound 48/80, a histamine-dependent pruritogen, and SLIGRL-NH2, a proteinase-activated receptor 2 agonist that induces histamine-independent acute itch [5,6]. Our results showed that compound 48/80 dramatically increased ERK activation in the spinal dorsal horn, whereas SLIGRL-NH2 did not do so (Figure 2C, D).

The proto-oncogene c-Fos is an indicator of neuronal activation in the central nervous system [20]. Obvious increases in the numbers of c-Fos-positive neurons were seen in the spinal dorsal horn 30 min after injection of histamine and chloroquine (Figure 2E, F). It should be noted that there were no significant differences between the two treatments in terms of number and location of c-Fos-positive cells (Figure 2E, F, H). Thus, the observed lack of pERK expression in chloroquine-treated mice is not due to any inability of the spinal neurons to respond to peripheral itch-related sensory inputs.

We next examined the distribution patterns of ERK activation in the spinal cord. Intradermal injections of histamine into the nape led to pERK expression in the lateral part of the bilateral cervical dorsal horns (Figure 2A), and intradermal injections of histamine into a single hind paw resulted in pERK activation in the medial part of the ipsilateral dorsal horn in the lumbar segments (Figure 2I). This topographical pattern of pERK activation in the spinal dorsal horn is consistent with the topographical termination sites of primary sensory axons from the body [21], thus further suggesting that ERK activation is specifically induced by histamine-related peripheral itch stimuli.

**Figure 3:**
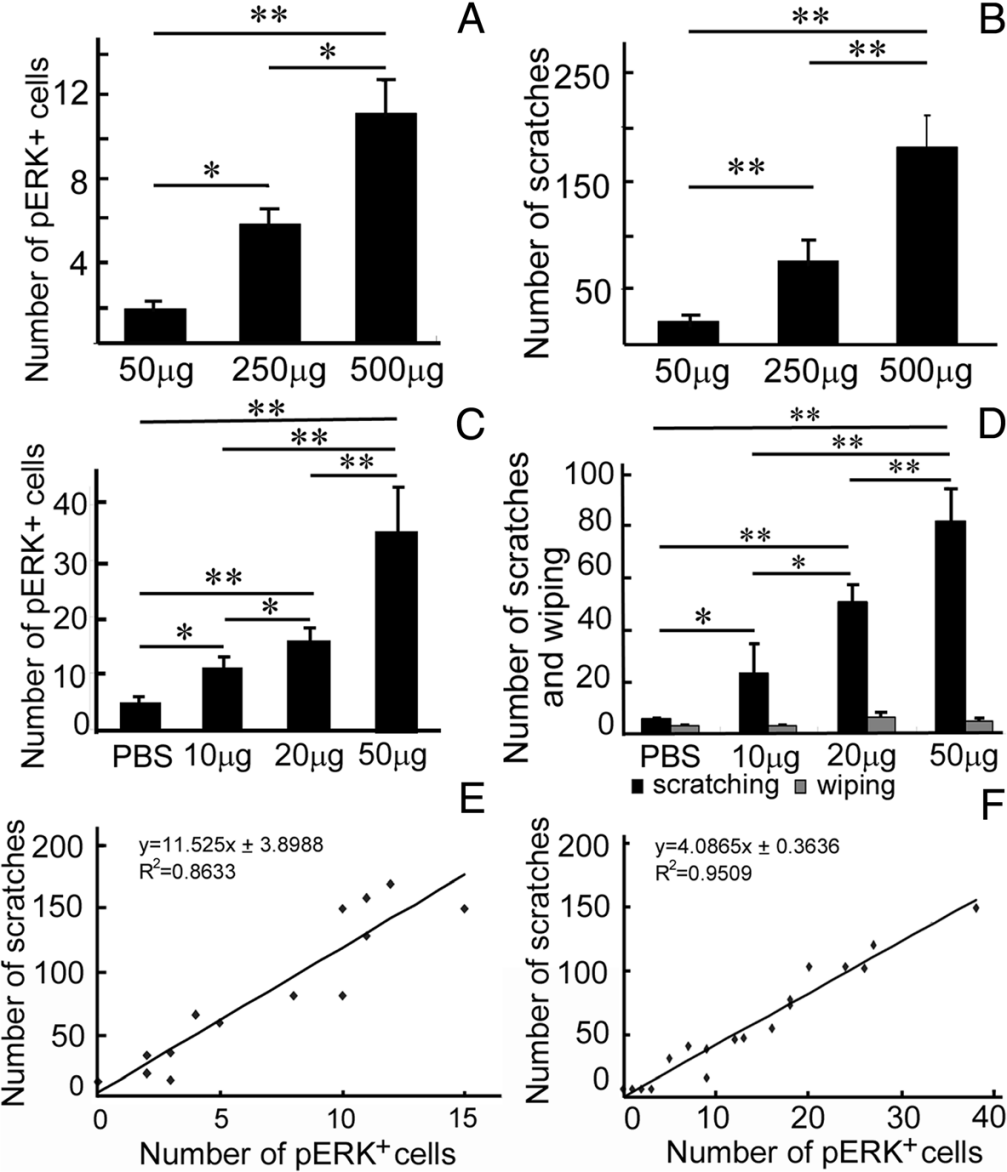
**Dose-dependent induction of pERK expression and scratching response by histamine. (A, B, E)** Thirty min after injection of histamine into the nape region, the number of pERK-positive cells in the spinal cord of mice treated with 500 μg of histamine was significantly increased compared with those treated with 50 μg or 250 μg **(A)**, and the number of scratches recorded in the first 30 min post-injection increased as the histamine dosage increased from 50 μg, to 250 μg to 500 μg **(B)**. One way ANOVA; *P* < 0.001; *n* = 6 for each group. The scratch numbers and ERK activation are highly correlated in the dose range from 50 to 500 μg **(E)**. Regression analysis; R^2^ = 0.86; *P* < 0.01; *n* = 5 for 50 μg and 500 μg groups and *n* = 4 for 250 μg group. **(C, D, F)** Thirty min post-injection of histamine into the cheek region, pERK-positive cells in the medullary dorsal horn **(C)** and scratches are increased in number in dose-dependent manner as the doses increased from 10 μg to 50 μg **(D)**. One way ANOVA; *P* < 0.001; n = 7 for PBS and 10 μg groups, n = 10 for 20 μg and 50 μg groups. Wiping number is maintained at a very low level with no obvious change. One way ANOVA; *P* > 0.05. Regression analysis of numbers of pERK-positive cells and scratches is shown in **(F)**. Regression analysis; R^2^ = 0.95, *P* < 0.01; *n* = 4 for PBS, 10 μg and 50 μg groups and *n* = 6 for 20 μg group. *P < 0.05; **P < 0.01.

To determine whether histamine-mediated ERK activation is dose dependent, we tested the effect of two lower doses of histamine (50 μg and 250 μg) in addition to the original dose of 500 μg. Treated mice were then sacrificed after 30-min behavioral observation and processed for pERK immunostaining. The results showed that lower doses of histamine also elicited scratching responses (Figure 3B) and pERK activation (Figure 3A), but to a significantly lesser degree than the 500 μg dose (Figure 3A, B). The scratch numbers and ERK activation induced by histamine were highly correlated in dose range (50–500 μg) used, as shown in Figure 3E. These results suggest that the activation of spinal ERK signaling by histamine is dose dependent.

### ERK signaling is also activated in histamine-induced itch cheek model

The itch cheek model can differentiate the behavior of itch and pain in mice, because histamine mainly elicits itch response shown by scratching with hind limb, while capsaicin evokes nociceptive response shown by wiping with the forelimb [22,23]. To further confirm the conclusion that ERK signals is activated by histamine-induced itch, histamine (10–50 μg in 10 μl PBS) was injected into the cheek region, and the behaviors of scratching and wiping were measured in 30 min after the injection. Histamine evoked vigorous scratching directed to the injection site, and scratching number was increased as the dose of histamine increased from 10 μg to 50 μg (Figure 3D). Although a small amount of wiping responses were observed, it did not have significant difference compared with PBS control or among groups treated with different doses of histamine (Figure 3D), which is in line with the previous report [23]. After the behavioral observation, the mice were sacrificed immediately and processed for pERK immunostaining as mentioned above. Many pERK-positive cells were observed in the superficial layers of the medullary dorsal horn in histamine-treated mice (Figure 2J), whereas only a few were found in controls injected with PBS. A significant difference was observed in pERK-positive cell number between histamine-injected and control mice as well as between histamine-injected mice with different doses (Figure 3C). Moreover, the increase of number of pERK-positive cells was highly correlated with the scratching response (Figure 3F). Thus, ERK activation is also present in histamine-induced itch cheek model. It should be noted that lower dose of histamine (e.g. 50 μg) could elicit robust scratches in itch cheek model but it only induced a small amount of scratches in itch nape model (Figure 3B, D). This may reflect the possibility that the orofacial region is more sensitive to histamine-induced itch possibly caused by a higher density of peripheral nerve innervation compared with the nape area.

### ERK signaling is activated in DNFB-induced itch

**Figure 4:**
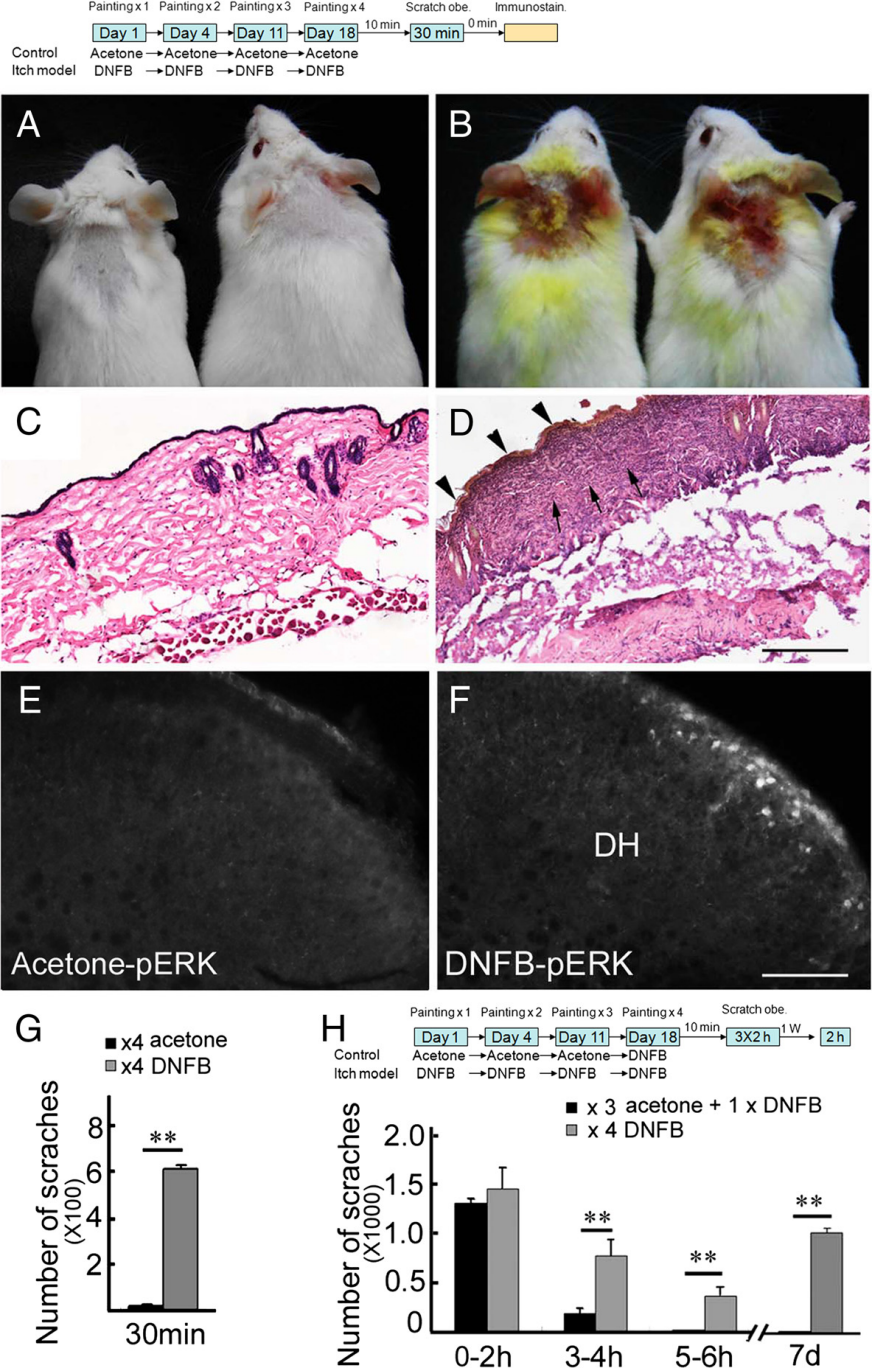
**pERK expression and scratches in DNFB-painted mice. (A, B)** Severe skin lesions are seen in mice treated with repeated DNFB painting **(B)**, but not in control mice with repeated acetone painting **(A)**. **(C, D)** Hematoxylin/eosin staining reveals irregular hyperplasia (triangles), enhanced staining of eosinophilic fibers and variable numbers of inflammatory cells (arrows) in the dermis of DNFB-treated skin samples **(D)**, but not in those treated with acetone alone **(C)**. **(E, F)** pERK is expressed in the dorsal horn of mice treated repeatedly with DNFB **(F)**, but not with acetone **(E)**. Comparison of scratches in 30 min between the two groups is shown in **(G)**. Student’s t-test; **P < 0.01; *n* = 5 for each group. The upper diagram indicates the detailed procedure for DNFB painting, behavior observation and immunostaining conducted in **(A-G)**. **(H)** Comparison of scratching number between the mice treated with 4 times of DNFB and those treated with 3 time of acetone and one time of DNFB. Significant differences are not seen in the first 2 h, but are present in the second and third 2 h as well as one week later. Two way repeated ANOVA; *P <* 0.01; One way ANOVA for comparison at individual time points; **P < 0.01; *n* = 5 for each group. The diagram shows the detailed procedure for DNFB and acetone painting, and behavior observation performed in **(H)**. DH, dorsal horn. Scale bars, 200 μm **(C, D)** and 100 μm **(E, F)**.

Consistent with the published report [24], repeat applications of DNFB to the skin of the nape resulted in scratching behaviors as well as lesions on the treated area (Figure 4A, B). Histological examination of DNFB-treated samples revealed a number of pathological alterations typical of atopic dermatitis, including irregular hyperplasia, enhanced staining of eosinophilic fibers and variable numbers of inflammatory cells (Figure 4C, D).

We next set out to establish whether ERK is also activated in the spinal cord in response to DNFB-induced itch. We observed a number of pERK-positive cells in the spinal cord of the treated mice (25.5 ± 1.58/section), whereas only a few positive cells were seen in control mice treated with acetone alone (1.6 ± 0.26/section; Figure 4E, F) 30 min after the last painting of DNFB. A significant difference in cell number between the two treatments was observed (*n* = 6 for each; Student t-test, *P* < 0.01). , showing that spinal ERK signaling is activated in DNFB-induced itch.

The scratching behavior was dramatically increased in the first 30 min in the DNFB-treated mice (Figure 4G). However, repeated-DNFB painting may lead to the acute allergic responses by DNFB, which contributes to the increased scratching behavior. To address this issue, we performed a control experiment in which mice painted with 3 times of acetone first and one time of DNFB last were included. These mice also showed vigorous scratching in the first 2 h, but it was largely attenuated in the second 2 h and disappeared in the third 2 h after the last painting DNFB (Figure 4H), suggesting that DNFB-elicited scratching is not due to an acute allergic response. Although there was no significant difference in scratching number in the first 2 h between the control mice and repeatedly DNFB-painted mice, the latter still showed a high level of scratching response in the second and third 2 h and even 1 week after the last DNFB painting (Figure 4H). Considering the appearance of typical pathological alterations of atopic dermatitis and long-lasting scratching behaviors, the repeated-DNFB painting protocol could be used to induce chronic itch.

### ERK activation is specifically localized to spinal neurons but not glia

**Figure 5:**
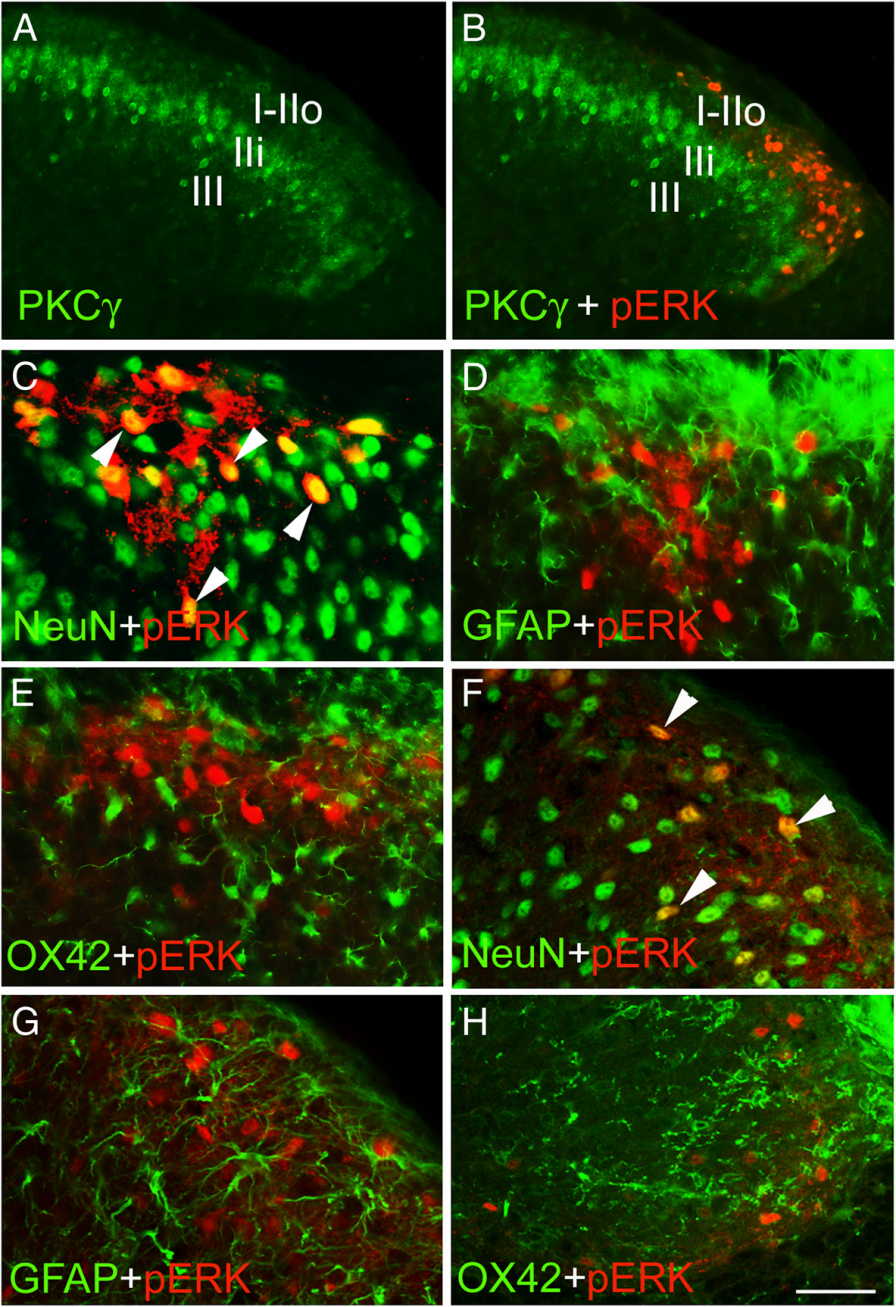
**Specific expression of pERK in the superficial spinal neurons in histamine-injected and repeated DNFB-painted mice. (A, B)** The vast majority of pERK-positive cells are located dorsally to the PKCγ-positive cells in histamine-inject mice. **(C-E)** pERK expression co-localizes with NeuN (arrowheads in **C**), but not with GFAP **(D)** or OX42 **(E)** in mice injected with histamine. **(F-H)** Laser confocal images show co-localization of NeuN and pERK (arrowheads in **F**), but no co-localization of pERK with GFAP **(G)** or Ox42 **(H)** in the spinal dorsal horn of repeated DNFB-painted mice. I, lamina I; IIi, inner lamina II; IIo, outer lamina II; III, lamina III. Scale bars, 100 μm **(A, B,)** and 50 μm **(C-H)**.

To further characterize the distribution of pERK in the spinal dorsal horn, we performed co-immunostaining of pERK and PKCγ, the latter of which is enriched in the inner lamina II [25]. We found that the vast majority of pERK-positive cells (120/134) were located dorsally to PKCγ-positive cells (Figure 5A, B), indicating that pERK expression is primarily localized in layer I and outer lamina II. To determine whether pERK is expressed in neurons or in glia, we co-immunostained pERK together with NeuN, GFAP or OX-42, which are specific makers for neuron, astrocyte and microglia, respectively. Thirty min after histamine injection, almost all pERK-expressing cells (180/195) were also positive for NeuN, but none of them were co-stained with GFAP (0/180) or OX-42 (0/190) antibody (Figure 5C-E). Similar results were obtained in DNFB-treated mice (Figure 5F-H). Taken together, these findings suggest that histamine- and DNFB-induced ERK activation occurs in spinal neurons, but not glia.

### Blocking phosphorylation of ERK suppresses histamine- and DNFB-induced itch responses

**Figure 6:**
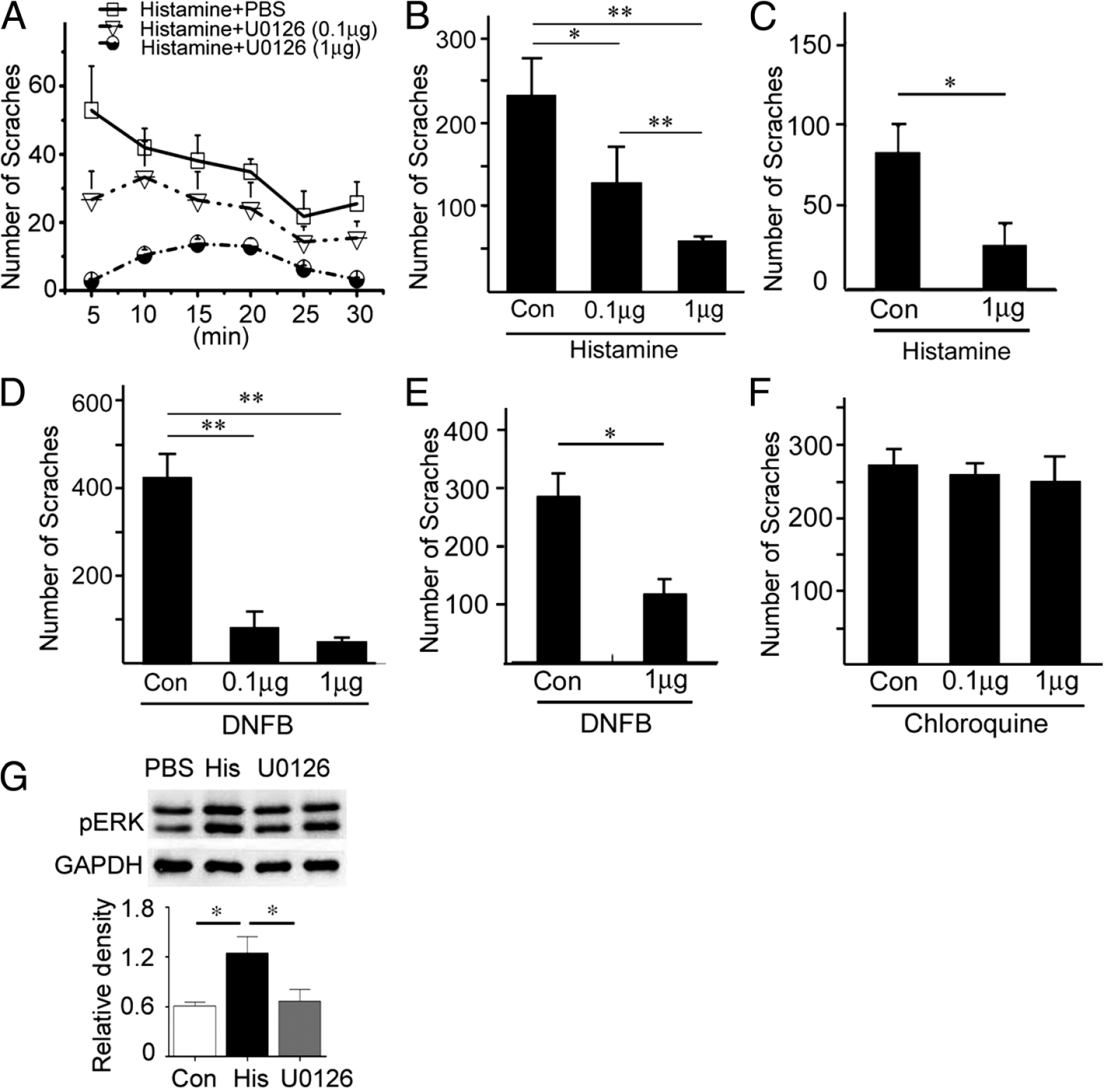
**Blocking ERK activation with U0126 attenuates the scratching behaviors induced by histamine and DNFB but not chloroquine. (A, B)** The number of scratches induced by histamine treatment (500 μg) is dramatically reduced by intrathecal administration of U0126 10 min before histamine injection **(A, B)**. The number of scratches in every 5 min is shown **(A)** and the total number in 30 min period is shown **(B)**. **(C)** The number of scratches induced by histamine treatment (500 μg) is dramatically reduced by intrathecal administration of U0126 10 min after histamine injection. **(D, E)** A similar effect by U0126 is also observed in repeated DNFB-painted mice. **(F)** Compared with controls, the number of scratches induced by chloroquine treatment (200 μg) is unchanged after intrathecal injection of U0126. In each group, at least 5 mice were used **(A-F)**. **(G)** Western blot shows the pERK expression in the spinal cord is increased in histamine-treated mice, but this increase was suppressed by the intrathecal administration of U0126. n = 3 for each group. One way ANOVA, for **A, B, D, F, G**; *P* < 0.01 in **A, B, D, G**; Student’s t-test for **C** and **E**; * *P* < 0.05; **P < 0.01).

The MEK inhibitor U0126, which inhibits the phosphorylation of ERK1/2 without affecting p38, JNK, or other MAP kinase pathway components [26], was used to study whether ERK activation is required for itch responses at the spinal level. U0126 (0.1 μg and 1 μg) was administered intrathecally [27] 10 min prior to intradermal pruritogen injection. We found that histamine-induced scratching behaviors were markedly inhibited by U0126 treatment in a dose-dependent manner (Figure 6A, B), but those induced by chloroquine remained unchanged (Figure 6F). Western blot data showed that the phosphorylation of ERK1/2 was indeed increased after the histamine injection, and it could be suppressed by the intrathecal application of U0126 (Figure 6G). In addition, intrathecal injection of U0126 10 min prior to the last DNFB application also dramatically inhibited the scratching response in repeatedly DNFB-treated mice (Figure 6D).

In order to know whether the ERK activation is involved in the maintenance of histamine-induced scratching response, the U0126 (1 μg) was intrathecally injected 10 min after histamine injection. Since the scratching has fully developed at 10 min post-histamine injection (Figure 6A), we chose this time point to explore this possibility. It showed that U0126 dramatically decreased the number of scratches 10 min after histamine injection (Figure 6C). Similar results were obtained in repeatedly DNFB-painted mice, as shown by an obvious reduction of scratching number when U0126 was applied 20 min after the last painting of DNFB (Figure 6E). These results indicate that ERK activation in the spinal cord is required for both of the initiation and maintenance of histamine- and DNFB-induced itch.

### H1 mediates histamine- but not DNFB-induced itch and ERK activation

**Figure 7:**
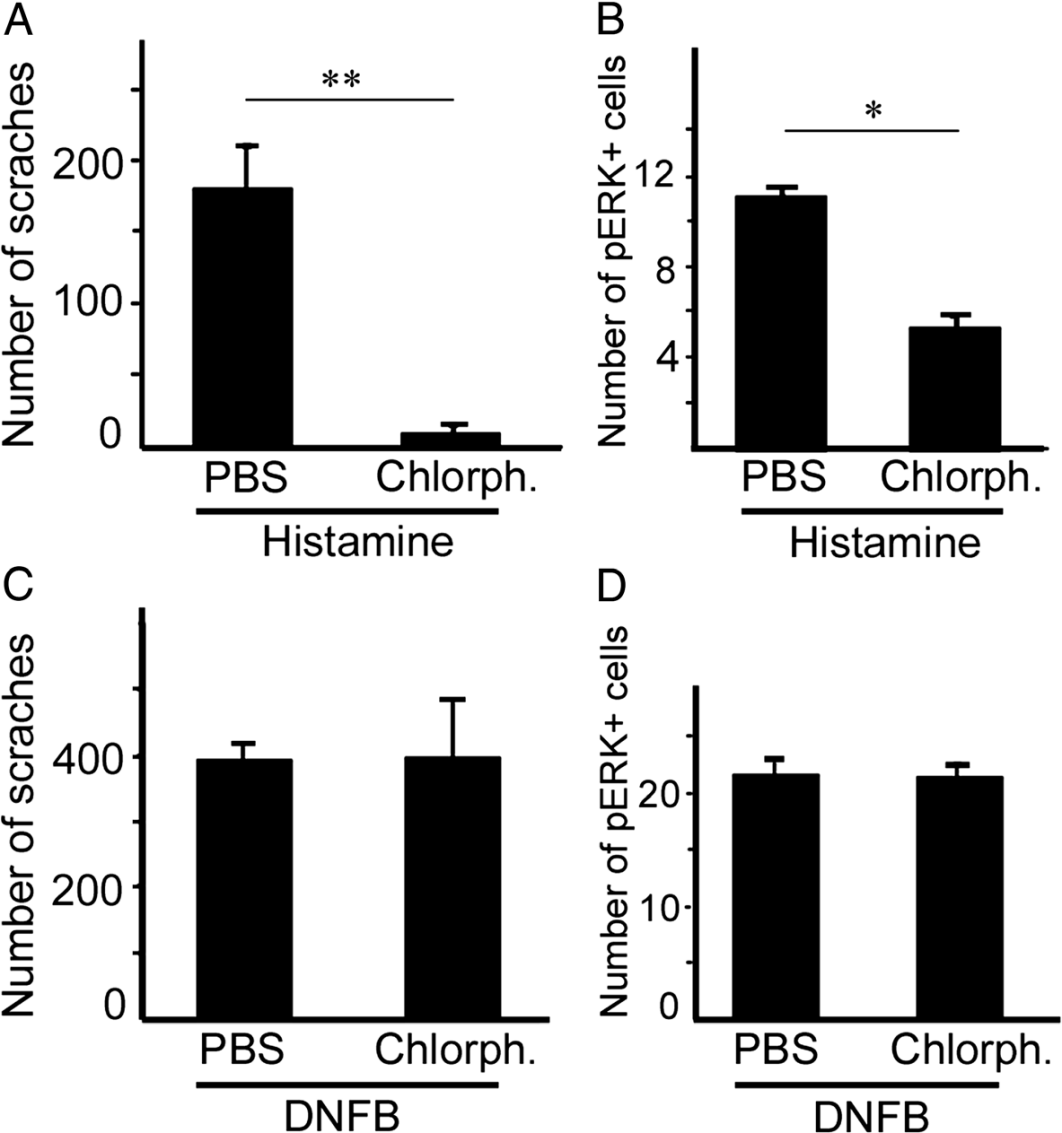
**Chlorpheniramine, an H1 antagonist, inhibits the scratching response and pERK expression induced by histamine but not DNFB. (A, B)** The numbers of scratches **(A)** and pERK-positive cells in the dorsal horn **(B)** induced by histamine injection are both significantly reduced after intraperitoneal administration of chlorpheniramine (10 mg/kg). **(C, D)** The number of scratches **(C)** and pERK-positive cells **(D)** induced by repeatedly painting DNFB remain unchanged in mice receiving the same chlorpheniramine treatment. Chlorph, chlorpheniramine. n = 6 for each group. Student’s t-test; *P < 0.05; **P < 0.01.

Histamine receptor 1 (H1) antagonists are widely used to alleviate allergic reactions and provide itch relief [2,28]. To establish whether histamine- and DNFB-induced ERK activation is mediated by the H1 receptor, its antagonist chlorpheniramine (10 mg/kg) was administered intraperitoneally 20 min prior to histamine injection or final DNFB application, and mice were sacrificed for pERK immunostaining after the 30-min itch response observation. Chlorpheniramine but not PBS treatment dramatically reduced histamine-induced scratching and ERK activation in the spinal cord (Figure 7A, B). On the other hand, neither DNFB-induced scratching nor pERK expression were affected by chlorpheniramine (Figure 7C, D), demonstrating that H1 receptor activation is not involved in DNFB-induced itch and spinal ERK activation.

Because chlorpheniramine (10 mg/kg) was administered intraperitoneally, and it may have sedative effects leading to reduced scratching in histamine-treated mice. We thus performed the following experiments. Open field test showed that application of chlorpheniramine in histamine-treated mice did not affect spontaneous locomotion activity (total traveled distance and velocity: 1385.53 ± 48.23 cm and 5.73 ± 0.83 cm/s in PBS group, respectively; 1273.36 ± 69.57 cm and 7.01 ± 0.91 cm/s in chlorpheniramine group, respectively. *n* = 5 for each; Student’s t-test, *P* > 0.05). Von Frey (withdraw threshold: 2.08 ± 0.23 g in PBS group and 2.23 ± 0.29 g in chlorpheniramine group, *n* = 5 for each; Student’s t-test, *P* > 0.05) and tail immersion tests (tail reflection latency at 48°C, 50°C and 52°C: 8.63 ± 0.40 s, 4.77 ± 0.16 s and 3.65 ± 0.23 s in PBS group; 8.58 ± 0.45 s, 5.03 ± 0.12 s and 3.45 ± 0.08 s in chlorpheniramine group, respectively. *n* = 5 for each; Student’s t-test, *P* > 0.05) showed that mechanical and thermal sensation were not changed compared with controls treated with PBS. Thus, it is unlikely that the reduction of scratching behaviors in histamine-treated mice is caused by the sedative effects of chlorpheniramine.

### Blocking activation of ERK inhibits enhanced neuronal activation induced by histamine and DNFB

**Figure 8:**
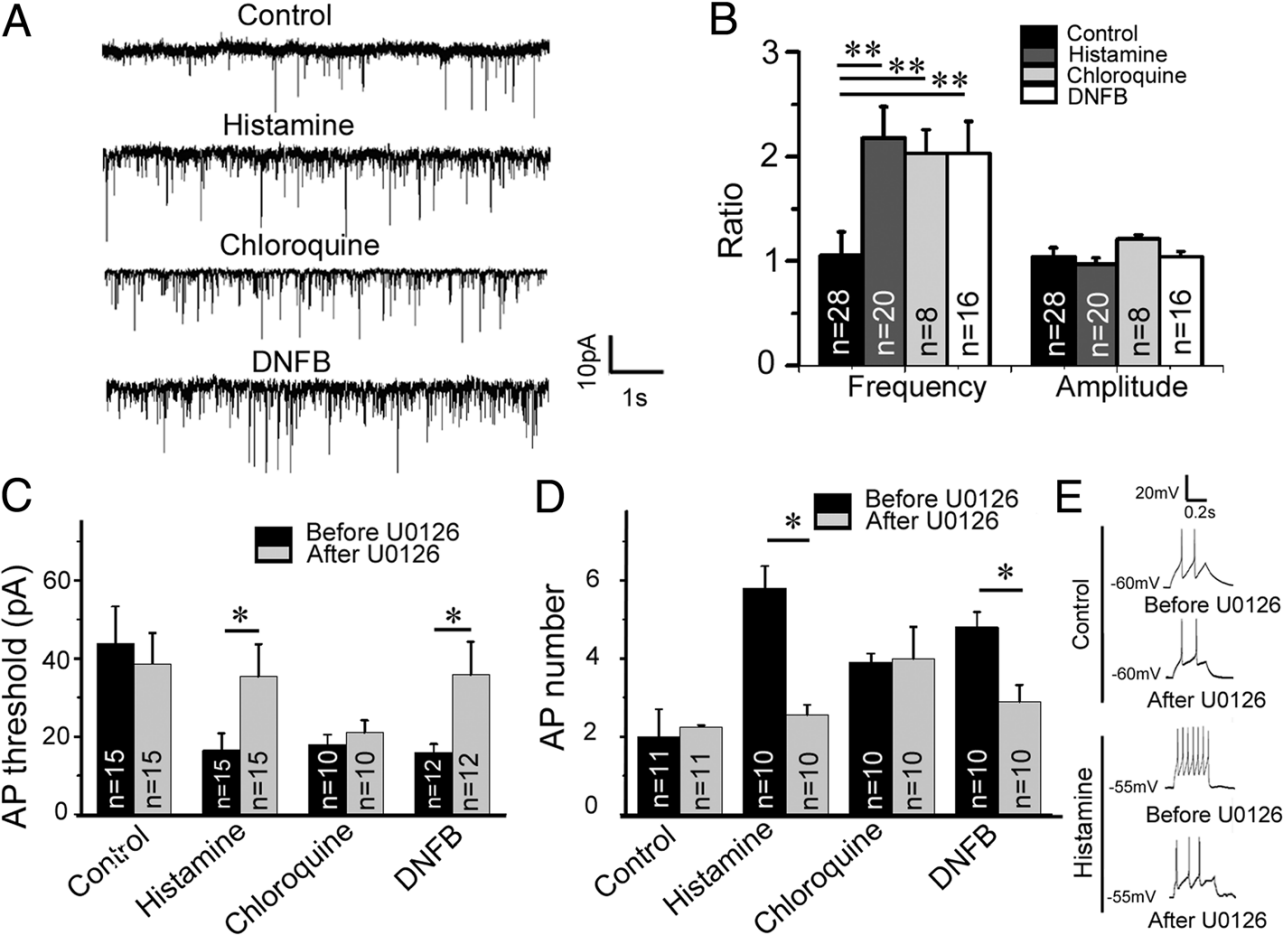
**Histamine- and DNFB-, but not chloroquine-induced high excitability of spinal dorsal horn neurons is suppressed by U0126.** Spinal slices were prepared 30 min after injection of PBS (20 μl), histamine (100 μg in 20 μl PBS), or chloroquine (80 μg in 20 μl PBS) into hind paw, or the last painting of DNFB on hind paw. **(A, B)** Voltage-clamp recording from outer laminar II neurons shows that the frequency but not the amplitude of sEPSC is increased in mice treated with histamine, chloroquine or DNFB. Six mice were used in each set of experiment, and recorded neuron numbers are shown in **(B)**. One way ANOVA, *P* < 0.01; **P < 0.01. **(C, D)** Current-clamp recording shows the decreased threshold of action potential **(C)** and increased frequency of action potential triggered by current injection **(D)** in histamine-, chloroquine- and DNFB-treated mice. Bath application of U0126 (1 μM) only prevents the changes in histamine- and DNFB-treated mice but not those treated with chloroquine. Five mice were used in each experiment and recorded neuron numbers are shown in **(C, D)**. Student’s t-test, *P < 0.05. **(E)** Samples of action potential frequency changes after bath application of U0126 in control mice and histamine-injected mice. AP, action potential.

To explore possible neurophysiological basis for ERK activation in itch sensation at the spinal level, patch-clamp recording was conducted in spinal slices prepared from histamine-, chloroquine- and DNFB-treated mice. We found that the frequency but not the amplitude of the spontaneous EPSC of spinal layer IIo neurons was significantly increased in histamine-, chloroquine- and DNFB-treated mice relative to controls (Figure 8A, B), suggesting more glutamate releasing in the central terminal of DRG neuron upon itch stimuli. In addition, the threshold of action potential was dramatically decreased and correspondingly frequency of action potential initiated by depolarizing current (40 pA, 400 μs) was significantly increased in the spinal dorsal horn neuron of histamine-, chloroquine- and DNFB-treated mice compared with PBS-treated controls (Figure 8C-E), suggesting that the excitability of spinal neurons is increased in these mice received itch-related sensory stimuli. Importantly, the enhanced excitability of spinal neurons was largely suppressed by bathing of U0126 in mice treated with histamine and DNFB, but not chloroquine (Figure 8C-E). Noted that neither the action potential threshold nor evoked action potential frequency from control mice receiving injection of PBS was changed by bath application of U0126 (Figure 8C, D). These results suggest that the activation of ERK is only involved in the elevation of spinal neuron excitability in histamine- and DNFB-treated mice but not those treated with chloroquine.

## Discussion

A large body of evidence has demonstrated that increased expression of pERK, a well-established indicator of ERK signaling activation, is involved in pain processing in the spinal cord [13,18,19,29-31]. In this study, we show for the first time that ERK activation is also required for itch sensation at the spinal level.

As mentioned above, ERK staining has been attributed to pain [13,20]. In this study, different doses of histamine (50–500 μg) were used to induce scratching response. However, it could be argued that the observed phosphorylation of ERK with high dose of histamine (e.g. 500 μg) administered at the nape region is due to histamine-produced nociceptive input rather than itch sensation. In addition, the rostral back injection of capsaicin also generates scratching behavior, although injection of capsaicin is usually considered to produce nociceptive sensations [23]. To further clarify these issues, itch cheek model was used, because itch and nociceptive responses can be represented by two distinct behaviors, scratching and wiping [23]. In histamine-injected mice, the scratching behavior, the indicator of itch, increased as the dose of histamine increased from 10 to 50 μg, while the wiping behavior, the indicator of nociception, was kept in a very low level and did not changed as the dose increased. In addition, pERK expression was found in the medullary dorsal horn, and pERK-positive cells were also increased as the dose of histamine increased. These results further support the idea that ERK signaling is activated by histamine-induced itch sensory input, although the nociceptive input-evoked spinal ERK activation could not fully excluded.

Acute itch is classified into histamine- and non-histamine-dependent categories, according to sensitivity to antihistamine treatment, suggesting that the molecular mechanisms underlying each category differ from one another [16,17]. A recent study demonstrated that the G-protein-coupled receptor MrgprA3 specifically mediates chloroquine-induced itch in DRG neurons, as evidenced by the finding that MrgprA3 mutant mice exhibit an overall reduction in scratching behaviors induced by chloroquine, but not histamine [7]. *In vivo* evidence also showed that histamine and cowhage (non-histamine pruritogen) activate distinct spinal dorsal horn neurons [32]. In this study, we reported herein that ERK signaling in the spinal dorsal horn is activated in response to histamine- but not chloroquine-induced acute itch. The observed lack of pERK expression in the chloroquine-treated mice is not due to any inability of the spinal neurons to respond to peripheral itch-related sensory inputs, because c-Fos expression levels and patterns in the spinal cord in histamine- and chloroquine-treated mice are comparable. Importantly, the specific MEK inhibitor U0126, attenuates histamine-induced itch responses as well as pERK expression in the spinal cord, thus confirming the specific role of ERK activation in histamine-induced acute itch.

What is the neurophysiological basis for ERK signals in itch sensation at the spinal level? ERK activation in the spinal dorsal horn only occurs in response to stimuli that are strong enough to cause C-fibers to fire [13], and histamine treatment can activate mechanically insensitive C-fibers [33]. Our results showed that the frequency of spontaneous EPSC of spinal dorsal horn neurons is increased, and the threshold of action potential is decreased leading to the increase of action potential frequency trigged by current injection in histamine-, chloroquine- and DNFB-treated mice. Based on these findings, we speculate that itch sensory input may elicit more glutamate releasing from the central terminal of primary sensory neurons thereby enhancing the excitability of spinal neurons. Our results also suggest that the intracellular ERK signaling pathway is involved in the elevation of spinal neuron excitability in histamine- and DNFB-induced but not chloroquine-induced itch, because the suppressive effect by bath application of U0126 was only observed in the mice treated with histamine and DNFB. However, there are some technical limitations in our patch-clamp recording. First, the recording was performed in spinal slices, and this *in vitro* condition may not fully reflect the nature of neuronal physiological property upon itch-related stimuli *in vivo*. Second, the timing difference among itch response, ERK activation and physiological recording may be an important factor affecting the outcome of patch-clamp recording. For example, histamine-induced itch responses are reduced 30 min and pERK activation is lowered 1 h, while the recording was performed at least 1.5 h after the injection of histamine due to the time spent in preparing the spinal slices. Thus, our recording may not truly reveal itch-induced physiological alteration of spinal neurons because it may change over time. *In vivo* recording is needed to further clarify this issue.

Chronic itch is a common symptom of several chronic diseases of the skin and other systems, such as atopic dermatitis and liver or kidney dysfunctions. This type of itch does not respond to treatment with anti-histamines and is therefore believed to be mediated by distinct molecular mechanisms [2,34]. In an attempt to elucidate some of these mechanisms, repeatedly applying DNFB to the skin was used, because it causes atopic dermatitis-like pathological alterations and vigorous scratching behaviors in mice [1]. Our data revealed that ERK signaling is activated in the spinal dorsal horn 30 min after the last painting of DNFB and that U0126 is able to suppress the scratching behaviors and enhanced excitability of spinal neurons induced by DNFB. However, a recent study reported that pERK-positive cells was not observed in the spinal cord 1 d after the last DNFB treatment, and intrathecal injection of U0126 did not affect itch response in DNFB-induced chronic itch in mice [35]. This may reflect the possibility that DNFB-induced phosphorylation of ERK is a transient event, but it triggers a long-lasting central plasticity leading to chronic itch response. They failed to reveal suppressive effect of U0126 in DNFB-treated mice may be due to different mouse strains used and experimental operations. As there is no effective treatment for chronic itch, ERK pathway may be a potential target for the development of new itch-relieving drugs. DNFB-induced itch last for at least one week after the last painting of DNFB, and more efforts should be paid in this time window for exploring the role of ERK signaling in chronic itch. In addition, our data also showed that the scratching behavior was observed in mice painted with one-time DNFB, suggesting that DNFB may be able to ignite the sensory fiber which transmits itch sensation.

A vast number of studies have conclusively shown that histamine-induced acute itch is mediated by the H1 receptor, which is the primary target for antihistamine treatment. Consistently, we found that blocking the H1 receptor with its antagonist chlorpheniramine effectively suppressed the scratching behaviors induced by histamine treatment. We also found that histamine-induced ERK activation is mediated by the H1 receptor, as illustrated by the finding that chlorpheniramine treatment significantly reduced pERK expression in histamine-treated mice. However, although chlorpheniramine dramatically decreased the scratching behavior, it only induced half reduction of ERK activation responded to histamine. We only tested this effect at one time point (20 min before histamine injection), and this may not be the most suitable time window to fully prevent ERK activation, and it is also likely that remaining ERK activation is caused by histamine-induced nociception that cannot be blocked by chlorpheniramine.

Chlorpheniramine has a sedative effect [36,37], which may contribute to the suppression of the scratching behaviors induced by histamine. However, the spontaneous locomotion, and mechanical and thermal sensations in chlorpheniramine-treated mice were not different from PBS-treated controls, suggesting the suppressive effect on histamine-induced itch response is less likely caused by the sedative effect of chlorpheniramine. This is also supported by the data that chlorpheniramine has no effect on scratching response in DNFB-treated mice. In DNFB-treated mice, neither of these suppression effects by chlorpheniramine was observed, indicating that the H1 receptor is not involved in DNFB-induced itch and spinal ERK activation, which is consistent with the clinical data [38]. Further studies are needed to identify which signaling pathway mediates DNFB-induced itch responses and ERK activation at the spinal level.

Itch and pain, although they share some similarities, are distinguished by unique behavioral responses [23], and believed to be mediated by distinct neural circuits [6]. In an rat model of neuropathic pain, spinal ERK activation is observed in neurons, astrocytes and microglia throughout the dorsal horn [31]. In contrast, in histamine-induced acute and DNFB-induced itch models used in the present study, pERK expression was restricted to spinal neurons primarily located in the superficial dorsal horn. Together, these findings support the idea that pain and itch are processed by different neuronal circuits in the spinal cord [16,17].

## Conclusions

Our data provide the first direct line of evidence that spinal ERK activation is involved in both histamine-dependent acute itch and DNFB-induced itch. Unlike histamine-induced itch, DNFB-induced itch and ERK activation is not mediated by the H1 receptor at the spinal level. The central role of ERK phosphorylation in spinal itch processing provides novel insight into the molecular mechanisms that govern itch generation and responses, from which novel anti-itch therapies may be developed.

## Materials and methods

### Animals

A total of 300 male adult ICR mice (25-35 g) were used. Animal care procedures were reviewed and approved by the Animal Study Committee at Tongji University School of Medicine, Shanghai, China.

### Drugs and administration

All pruritogens were purchased from Sigma-Aldich (St. Louis, MO). Histamine (H7125; 500 μg), chloroquine (C6628; 200 μg), compound 48/80 (C2313; 100 μg) and proteinase-activated receptor 2 agonist SLIGRL-NH2 (S9317; 100 μg) were dissolved in 50 μl of 0.01 M phosphate buffered saline (PBS; pH7.4) and injected intradermally into the nape to generate acute itch responses [5,6]. In addition, histamine (200 μg in 20 μl PBS) was also injected intraplantarly into the hind paw on one side, in order to explore the topographical expression of pERK in the spinal cord. To induce itch responses in the facial region (itch cheek model), histamine (10–50 μg) dissolved in 10 μl of PBS was injected intradermally into the cheek region as reported previously [22,23]. It should be noted that the mice were not anesthetized during the injection of pruritogens as described previously [23]. To exclude the possibility that pERK is elicited by scratch stimuli rather than histamine, pERK expression was also examined in sodium pentobarbital-anesthetized mice (40 mg/kg body weight, i.p.) that did not scratch.

To induce atopic dermatitis, 150 μl of 0.15% DNFB (D1529; Sigma-Aldich) in acetone was repeatedly applied to the shaved skin of the nape [1,24]. DNFB was applied twice in the first week and once per week for the next two weeks, for a total of four applications. Control mice were administered the same treatment, but either painted with four times of acetone alone or with 3 times of acetone first and one time of DNFB last on the day behavioral observation commenced.

The MEK inhibitor U0126 [1,4-diamino-2,3-dicyano-1,4-bis (oaminophenylmercapto) butadiene] (U120; Sigma-Aldich) was dissolved in dimethyl sulfoxide (DMSO) to create a 100X stock solution, which was stored at −80°C and diluted in PBS prior to use. U0126 (0.1 μg and 1 μg in 10 μl PBS) was administered intrathecally with a 30 gauge needle at the lumbar level, as described previously [39], 10 min prior to pruritogen injection or final DNFB application. In order to explore if ERK activation is also involved in the maintenance of itch response, U0126 was also applied 10 min after histamine intradermal injection or 20 min after the last painting of DNFB. Control mice received an intrathecal injection of the same volume of 1% DMSO in PBS.

The histamine H1 receptor antagonist chlorpheniramine (C3025; Sigma-Aldich; 10 mg/kg; dissolved in PBS) was administered intraperitoneally 20 min prior to pruritogen injection or final DNFB application. Control mice received an equal volume of PBS only.

### Behavior study

Itch response evaluation was performed as described previously [5,23]. For acute itch, mice were habituated to ambient conditions for 30 min and then injected intradermally in the nape or cheek with one of the pruritogens mentioned above. Hind paw scratching behaviors directed towards injection site were recorded for 30 min immediately following the injection. In DNFB-treated mice, scratching behaviors were evaluated in the same way starting at 10 min after the last application. For some experiments, the scratching behavior was recorded uninterruptedly for 6 h immediately after and for 2 h one week after the last painting of DNFB.

The following behavioral observation was used to examine whether chlorpheniramine used in our study show sedative effects, as this may affect scratching response induced by histamine and DNFB. For the open field test, the mice were put into the center of the open chamber (42 cm wide × 42 cm high × 42 cm long) 20 min after intraperitoneal injection of chlorpheniramine (10 mg/kg). Mice injected with PBS were used as control. The chamber was thoroughly cleaned up with 75% ethanol between tests. During the 30-min test session, the total distance and the velocity of movement were recorded by camcorders and analyzed with ehtoVision XT8 (Noldus Information Technology, Wageningen, Netherlands). For the von Frey test, mice were put on the elevated wire grid for habituation for 2 h and the plantar surface of the hind paw was stimulated with calibrated von Frey monofilaments. The 50% paw withdrawal threshold for the von Frey assay was determined using Dixon’s up-down method [40]. For the tail immersion test, mice were gently restrained in a black cloth pocket which the mice voluntarily entered. The protruding one third of the tail was then dipped into the water at 48°C, 50°C and 52°C, respectively, with 15 min interval. Latency to respond to the heat stimuli with vigorous flexion of the tail was measured three times and averaged. In all behavioral tests, the behavioral observer was blind to experimental designs.

### Immunohistochemical and histological analyses

Mice were deeply anesthetized with sodium pentobarbital and perfused through the ascending aorta with PBS followed by 4% paraformaldehyde in 0.1 M phosphate buffer (pH 7.4). After the perfusion, spinal cord and DNFB-treated nape skin were removed and post-fixed overnight. For mice received injection of pruritogens in the nape region, cervical cord segments (C4-C8) were removed; for those received injection of histamine in the hind paw, lumbar cord segments (L4-L5) were removed; for those received the injection in cheek region, the caudal hindbrain was removed.

Samples were cut transversely into 30 μm-thick frozen sections on a cryostat. Every sixth section was collected as one set and they were processed for immunofluorescence. The following primary antibodies: rabbit anti-pERK1/2 antibody (1:600; Cell Signaling Technology, Danvers, MA), rabbit anti-c-Fos antibody (1;1000; Santa Cruz Biotechnology Inc, Santa Cruz, CA), mouse anti-NeuN antibody (1:1000; Millipore, Temecula, CA), mouse anti-OX42 antibody (1:500; Serotec, Oxford, UK) and mouse anti-GFAP antibody (1:1000; Millipore). Sections were incubated with the primary antibodies overnight at 4°C, and then with Cy3- (1:500, Jackson ImmunoResearch, West Grove, PA) or Alexa Fluor 488-conjugated species-specific secondary antibodies (1:500; Invitrogen, Carlsbad, CA) for 2 h at room temperature. For double immunofluorescence, sections were incubated with a mixture of rabbit polyclonal and mouse monoclonal primary antibodies, followed by a mixture of Alexa Fluor 488- and Cy3-conjugated anti-rabbit and anti-mouse IgG. In addition, hematoxylin/eosin staining was performed on sections of DNFB- and acetone-treated skin to identify any pathological alterations.

For pERK and PKCγ co-immunostaining (both are rabbit polyclonal antibodies), we employed the tyramide signal amplification system (TSA Plus Biotin Kit, PerkinElmer, Waltham, MA) [41,42]. Spinal cord sections were first incubated overnight at 4°C with rabbit anti-pERK antibody (1:15000), a dilution at which no immunoreactivity for pERK could be detected by the conventional staining procedure. Sections were then incubated with HRP-labeled goat anti-rabbit IgG (1:100; KangChen, Shanghai, China) for 3 h and TSA Biotin Amplification Reagent (1:50; PerkinElmer) for 10 min at room temperature. Signals were visualized with Cy3-conjugated streptavidin (1:500; PerkinElmer). After being washed in PBS, sections were incubated with the second primary antibody, rabbit anti-PKCγ antibody (1:1000; Santa Cruz Biotechnology), overnight at 4°C, and then with Alexa Fluor 488-conjugated donkey anti-rabbit IgG (1:500; Invitrogen) for 3 h at room temperature.

The stained sections were observed under a Nikon fluorescence microscopy equipped with a Nikon Coolpix digital camera (DS-Ri1; Tokyo, Japan) or laser confocal microscopy (TCS SP5 II; Leica, Germany). For counting pERK-positive cells in the cervical, lumbar and medullary dorsal horns, all immunostained sections in the one set of sections were included. A cell was considered to be pERK positive when signal/noise ratio (intensity of immunofluorescence in cell body vs background) was > 2. All images were made into figures using Adobe Photoshop (Adobe Systems Incorporated, San Jose, CA) and only minor adjustments to the contrast and brightness settings were applied if necessary.

### Western blot

To confirm the suppressive effects of intrathecal U0126 on spinal phosphorylation of ERK, we performed Western blot in mice received histamine (500 μg) injection at the nape region. U0126 (1 μg) was applied 10 min before the injection, and cervical cord segments were removed 10 min after the injection. They were homogenized in the T-PER Tissue Protein Extraction Reagent with 10% v/v Halt™ proteinase and phosphatase inhibitor cocktail (Thermol Scientific, Amarillo, TX). After a centrifugation of 12000 rpm for 15 min at 4°C, the supernatant was collected, boiled with protein loading buffer in 95°C for 5 min and subjected to SDS-PAGE electrophoresis. The cellulose nitrate membrane contained the target protein was incubated with the pERK primary antibody (1:1000; Cell Signaling Technology) overnight in 4°C, with the horseradish peroxidase-conjugated secondary antibody for 1.5 h at room temperature, and visualized with the Luminata™ Crescendo western HRP substrate purchased from Millipore (Billerica, MA).

### Spinal slice preparation

Mice (4–6 week old) received intraplantar injection of histamine (100 μg in 20 μl PBS), chloroquine (80 μg in 20 μl PBS) or PBS (20 μl) were used. For DNFB-treated mice, DNFB were repeatedly painted on one hand paw as mentioned earlier. The lumbar spinal cord (L4-L5) was removed from the mice under urethane anesthesia as reported previously [43-45]. Spinal segment was placed in a shallow groove formed in an agar block and glued to the bottom of the microslicer stage. Transverse slices (600 μm) were cut on a vibrating microslicer. The slices were perfused with Kreb’s solution (3 ml/min) saturated with 95% O_2_ and 5% CO_2_ at 36 ± 1°C for 1–2 h prior to experiment. The Krebs solution contains (in mM): NaCl 117, KCl 3.6, CaCl_2_ 2.5, MgCl_2_ 1.2, NaH_2_PO_4_ 1.2, NaHCO_3_ 25 and glucose 11.

### Patch clamp recordings in spinal slices

The whole cell patch-clamp recordings were made from outer lamina II neurons in voltage and current mode. Patch pipettes were fabricated from thin-walled, borosilicate, glass-capillary tubing (1.5 mm o.d., World Precision Instruments, Sarasota, FL). After establishing the whole-cell configuration, neurons were held at −70 mV for recording sEPSC in voltage clamp mode or action potential in current clamp mode, respectively. The resistance of a typical patch pipette was 5–10 MΩ. The internal solution contains (in mM): potassium gluconate 135, KCl 5, CaCl_2_ 0.5, MgCl_2_ 2, EGTA 5, HEPES 5 and ATP-Mg 5. The signals were amplified with an Axopatch 700B amplifier (Molecular Devices, Sunnyvale, CA) and filtered at 2 kHz and digitized at 5 kHz. All data were stored with a personal computer using pCLAMP 10 software and analyzed with Mini Analysis 6.0 program (Synaptosoft, Decatur, GA) and Clampfit.

### Quantification and statistics

Data are expressed as mean ± SEM. Differences between two groups were compared using 2-tailed Student’s t-test. One way ANOVA followed by a Fisher post hoc test to evaluate the differences among multiple groups. Two way repeated ANOVA was used for comparison of data from the mice with repeatedly painting of DNFB or acetone, because these measurements were collected in the same mice at different time points, as shown in Figure 4H. The regression analysis was used to expression the correlation of the ERK activation and the number of scratches. All the data were analyzed using OriginPro 8.5 (OriginLab, Northampton, MA). The criterion for statistical significance was *P* < 0.05.

## Abbreviations

AP: Action potential; Chloph: Chlorpheniramine; df: Dorsal fasciculus; DH: Dorsal horn; DMSO: Dimethyl sulfoxide; DNFB: 2,4-dinitrofluoroenzene; DRG: Dorsal root ganglia; ERK: Extracellular signal-regulated kinase; GRPR: Gastrin releasing peptide receptor; lf: Lateral fasciculus; MDH: Medullary dorsal horn; pERK: Phosphorylation of extracellular signal-regulated kinase; sEPSP: Spontaneous excitatory postsynaptic current; t: Spinal trigeminal tract; U0126: 1,4-diamino-2,3-dicyano-1,4-bis (oaminophenylmercapto) butadiene; IIi: Inner lamina II; IIo: Outer lamina II; III: Lamina III.

## Competing interests

The authors declare that they have no competing interests.

## Authors’ contribution

LZ and YQD designed the research and wrote the paper. LZ and GYJ carried out the immunostaining, behavior test and analyzed the data. GYJ performed the western blot experiment and analyzed the data. LZ performed the electrophysiology experiment and analyzed the data. NJS performed the hematoxylin/eosin staining of skin and analyzed the pathological alteration. JYC,YH and QXW participated in the preparation of samples. All authors read and approved the final manuscript.
